# βENaC is a molecular component of a VSMC mechanotransducer that contributes to renal blood flow regulation, protection from renal injury, and hypertension

**DOI:** 10.3389/fphys.2012.00341

**Published:** 2012-08-28

**Authors:** Heather A. Drummond

**Affiliations:** Department of Physiology and Biophysics, University of Mississippi Medical CenterJackson, MS, USA

**Keywords:** baroreflex, degenerin, epithelial Na^+^ channel, hypertension, mechanotransduction, myogenic constriction, renal blood flow, renal injury

## Abstract

Pressure-induced constriction (also known as the “myogenic response”) is an important mechano-dependent response in certain blood vessels. The response is mediated by vascular smooth muscle cells (VSMCs) and characterized by a pressure-induced vasoconstriction in small arteries and arterioles in the cerebral, mesenteric, cardiac, and renal beds. The myogenic response has two important roles; it is a mechanism of blood flow autoregulation and provides protection against systemic blood pressure-induced damage to delicate microvessels. However, the molecular mechanism(s) underlying initiation of myogenic response is unclear. Degenerin proteins have a strong evolutionary link to mechanotransduction in the nematode. Our laboratory has addressed the hypothesis that these proteins may also act as mechanosensors in certain mammalian tissues such as VSMCs and arterial baroreceptor neurons. This article discusses the importance of a specific degenerin protein, β Epithelial Na^+^ Channel (βENaC) in pressure-induced vasoconstriction in renal vessels and arterial baroreflex function as determined in a mouse model of reduced βENaC (βENaC m/m). We propose that loss of baroreflex sensitivity (due to loss of baroreceptor βENaC) increases blood pressure variability, increasing the likelihood and magnitude of upward swings in systemic pressure. Furthermore, loss of the myogenic constrictor response (due to loss of VSMC βENaC) will permit those pressure swings to be transmitted to the microvasculature in βENaC m/m mice, thus increasing the susceptibility to renal injury and hypertension.

## Introduction

Mechanotransduction in vascular tissues is a topic of physiologic and pathophysiologic relevance. Chronic and transient mechanical forces contribute to development of atherosclerosis, angiogenesis, endothelial function, ischemia-reperfusion injury, myogenic constriction, and hypertension (Hill et al., 2006; Shin et al., 2008; Heo et al., 2011; Johnson et al., 2011). However, the molecular mechanisms underlying transduction of mechanical forces, particularly the transduction of transient mechanical forces into rapid changes in cellular function, remain unclear. Our laboratory has been investigating the molecular mechanism(s) underlying initiation of the myogenic response in renal vasculature.

## What is the myogenic response and why is it important?

### The myogenic response

**Figure 1:**
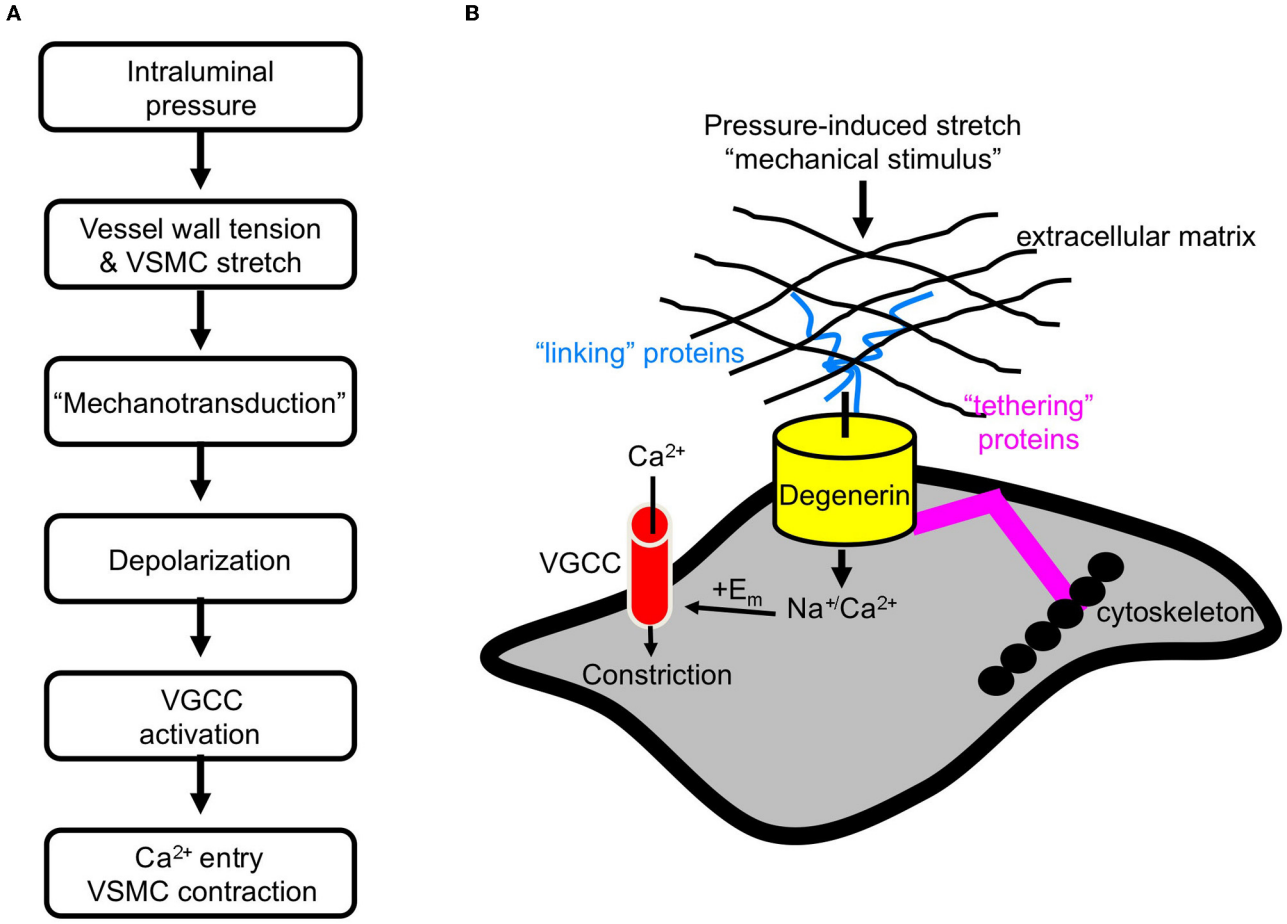
**Initiation of the myogenic response. (A)** The sequence of events leading myogenic constriction. **(B)** Hypothetical role of degenerin proteins as a mechanotransducer in VSMCs.

The myogenic response was initially described over 100 years ago (Bayliss, 1902). It is generally accepted that the myogenic response is initiated by pressure-induced vessel wall stretch (Davis and Hill, 1999; Hill et al., 2001). Expansion of the vessel wall stretches vascular smooth muscle cells (VSMCs) circumferentially arranged around the vessel. In turn, VSMC stretch initiates a depolarization event, which is thought to activate voltage gated Ca^2+^ channels (VGCC). Ca^2+^ channel activation stimulates Ca^2+^ influx and triggers vasoconstriction (Figure 1A) (Davis and Hill, 1999; Hill et al., 2001).

### Physiological significance of myogenic constriction: regulation of renal blood flow and protection from injury

The myogenic response is important because it participates in two processes. First, it is a mechanism of blood flow autoregulation, in which blood flow is tightly controlled despite changes in systemic perfusion pressure (Navar, 1978; Navar et al., 1982). Myogenic constriction is fast acting and adjusts vascular resistance to a change in perfusion pressure within 5–10 s. The other mechanism, tubuloglomerular feedback (TGF), is slower adjusting vascular resistance within 6–25 s (Cupples et al., 1996; Just and Arendshorst, 2003; Cupples and Braam, 2007; Just and Arendshorst, 2007). The fast nature of the myogenic response has led several investigators to suggest the other purpose of the myogenic response is to prevent the transmission of high systemic pressures to the fragile glomerular microvasculature, thus protecting microvasculature from pressure-related injury associated with hypertension, diabetes, and end stage renal disease (Loutzenhiser et al., 2002; Marsh et al., 2005). While much is understood about signaling mechanisms underlying VSMC contraction, our understanding of the signaling mechanisms that transduce changes in intraluminal pressure into a cellular signaling event, i.e., the events that *initiate* myogenic constriction, is limited.

Several signaling mechanisms are essential to the transduction of mechanical stimuli including, but not limited to, transient receptor potential (TRP) channels, integrins, membrane-associated lipids, VGCC, and *potassium channels* (Davis and Hill, 1999; Davis et al., 2001; Hill et al., 2001, 2006; Montell, 2005). Excellent reviews on these topics can be found elsewhere (Davis and Hill, 1999; Davis et al., 2001; Hill et al., 2001, 2006; Montell, 2005). However, in addition to these mechanisms, we hypothesize degenerin proteins are also essential to VSMC mechanotransduction by acting as components of a large, heteromultimeric mechanosensor that transduces stretch into a cellular event. We do not hypothesize that degenerin proteins form “the vascular mechanosensor,” but rather they are components of a large mechanosensing complex that includes, or is closely associated with, other signaling mechanisms such as integrins, TRP channels, VGCC, and membrane associated lipids. Although studies addressing this latter point have not been published, this review addresses numerous studies from our laboratory supporting an essential role for at least one degenerin protein in VSMC mechanotransduction.

## Could degenerin proteins participate in mechanotransduction in VSMCs?

### Degenerin proteins

Degenerin proteins are a large family of proteins expressed in a diverse species, including the nematode, *Caenorhabditis elegans* (*C. elegans*), *Drosophila*, and mammals, with strong evolutionary ties to mechanotransduction in neuronal and muscle tissues (Kizer et al., 1997; Tavernarakis and Driscoll, 1997, 2001; Benos and Stanton, 1999; Mano and Driscoll, 1999; Trujillo et al., 1999; Kellenberger and Schild, 2002; Goodman and Schwarz, 2003; Syntichaki and Tavernarakis, 2004; Arnadottir and Chalfie, 2010). Members of this family share a common structure: intracellular N- and C-termini and a large extracellular domain separated by two membrane-spanning domains. Many of the degenerin proteins form homo- and heteromultimeric, non-voltage gated, Na^+^/cation channels (Benos et al., 1995; Tavernarakis and Driscoll, 2001; Kellenberger and Schild, 2002; Goodman and Schwarz, 2003).

In mammals, three subfamilies of degenerin proteins have been identified: the Epithelial Na^+^ Channel (ENaC), Acid Sensing Ion Channel (ASIC), and Brain Liver Intestine Na^+^ Channel (BLINaC) proteins. ENaC proteins are known for their role in Na^+^ and water transport in the kidney, lung, and colon epithelia. In these tissues, α, β, and γENaC proteins form a non-voltage gated, Na^+^ selective ion channel. The αβγENaC channel is inhibited by low doses (submicromolar to low micromolar range) of the diuretic amiloride and its analog benzamil. ENaC proteins have also been found at several important sites of mechanotransduction including light touch receptors in skin (Pacinian Corpuscles, Meissner Corpuscles, Merkel Cells, free nerve endings), osteoclasts, keratinocytes, arterial baroreceptor neurons, endothelial cells, and VSMCs (Kizer et al., 1997; Drummond et al., 2000, 2001, 2004; Mauro et al., 2002; Wang et al., 2009). Furthermore, αβγENaC channels are gated by shear stress (Satlin et al., 2001; Carattino et al., 2004). Because of their close evolutionary relationship to the *C. elegans* degenerins, localization in mechanosensitive tissues, and ability to form ion channels that may be gated by mechanical forces, ENaC proteins have been considered as likely components of mechanosensitive ion channel complexes in vertebrate tissue.

### The degenerin mechanosensor: a potential model for a mammalian mechanosensor

A model of a mammalian mechanosensor has not been established. However, numerous genetic studies have led to the development of an “all-purpose” model of mechanotransducers in *C. elegans* neuronal and muscle tissue (Syntichaki and Tavernarakis, 2004). The model consists of three components: (1) an ion-conducting pore, (2) extracellular matrix and proteins that may link the pore to the matrix, and (3) cytoskeleton and proteins that may link the pore to the cytoskeleton. In this model, degenerin proteins form the ion channel pore. The application of a mechanical force is transduced through the extracellular matrix to gate the channel. Thus, the interaction between the pore forming degenerin proteins and the extracellular matrix is considered critical to channel gating. The cytoskeleton may also participate in transduction of the applied force and along with other extracellular proteins, may also stabilize the pore forming proteins at the cell surface. We hypothesize that a similar model applies to mechanotransduction in mammalian tissues. Therefore, we are using the *C. elegans* model as a platform to develop a model of a mammalian mechanosensor (Figure 1B). We further hypothesize that mammalian degenerin proteins form the ion-conducting pore. Activation of the mechanosensor leads to influx of Na^+^ and/or Ca^2+^, which leads to membrane depolarization and subsequent activation of VGCC.

## The early years: establishing a role for degenerin proteins in renal myogenic constriction

### ENaC proteins in renal VSMCs

**Figure 2:**
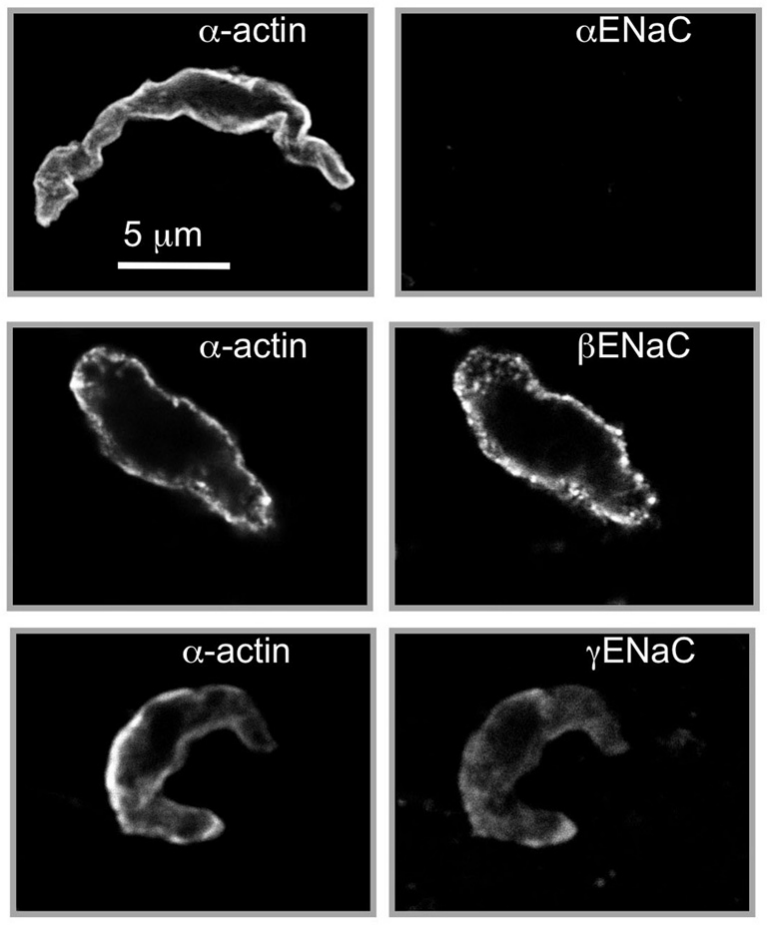
**Localization of βENaC and γENaC in enzymatically dissociated renal VSMCs.** VSMCs were identified by labeling with a smooth muscle actin (left column). We detected βENaC and γENaC, but not αENaC, at or near the VSMC surface. The strongest labeling is consistently observed for βENaC. Scale bar represents 5 μm. (Figure includes images reproduced from American Journal of Physiology, Renal Physiology 289; F891–F901, 2005, Figure 2).

To consider ENaC proteins as mechanosensors mediating pressure-induced constriction in blood vessels, ENaC proteins must be expressed in VSMCs and located at the site of mechanotransduction, near the cell surface. Therefore, early studies focused on the expression and localization of ENaC proteins in VSMCs isolated from myogenically active vascular beds (Drummond et al., 2004; Jernigan and Drummond, 2005; Jernigan et al., 2008). As shown in Figure 2, VSMCs enzymatically dissociated from renal arterial segments express β and γENaC, but not α, at or near the cell surface membrane (Jernigan and Drummond, 2005). Since the expression level of the *C. elegans* degenerin proteins is low (Lai et al., 1996), we expected a similar low expression level in VSMCs, and thus extensive optimization of the immunofluorescence assay was required. The localization pattern is significant because the cell surface is the site where the mechanosensor might be predicted to be located. The lack of αENaC in VSMCs is also an important finding. It has been suggested that the lack of αENaC would render β and γENaC unable to form a channel in VSMCs. While αENaC is required for high conductance and constitutive activity properties of the “classical” ENaC channel found in epithelial tissue, β and γ–rat ENaC can form an amiloride-sensitive, Na^+^ conducting channel in the absence of αENaC (Bonny et al., 1999). The presence of δENaC, a subunit that can confer a high level of activity to βγENaC in the absence of αENaC, has not yet been determined as it is apparently not present in mouse or rat (Waldmann et al., 1995; Kapoor et al., 2009; Giraldez et al., 2012). The possibility of another subunit, such as an ASIC protein or unidentified ENaC, interacting with β and γENaC to form a channel has not been ruled out.

### ENaC inhibition suppresses renal myogenic constriction

**Figure 3:**
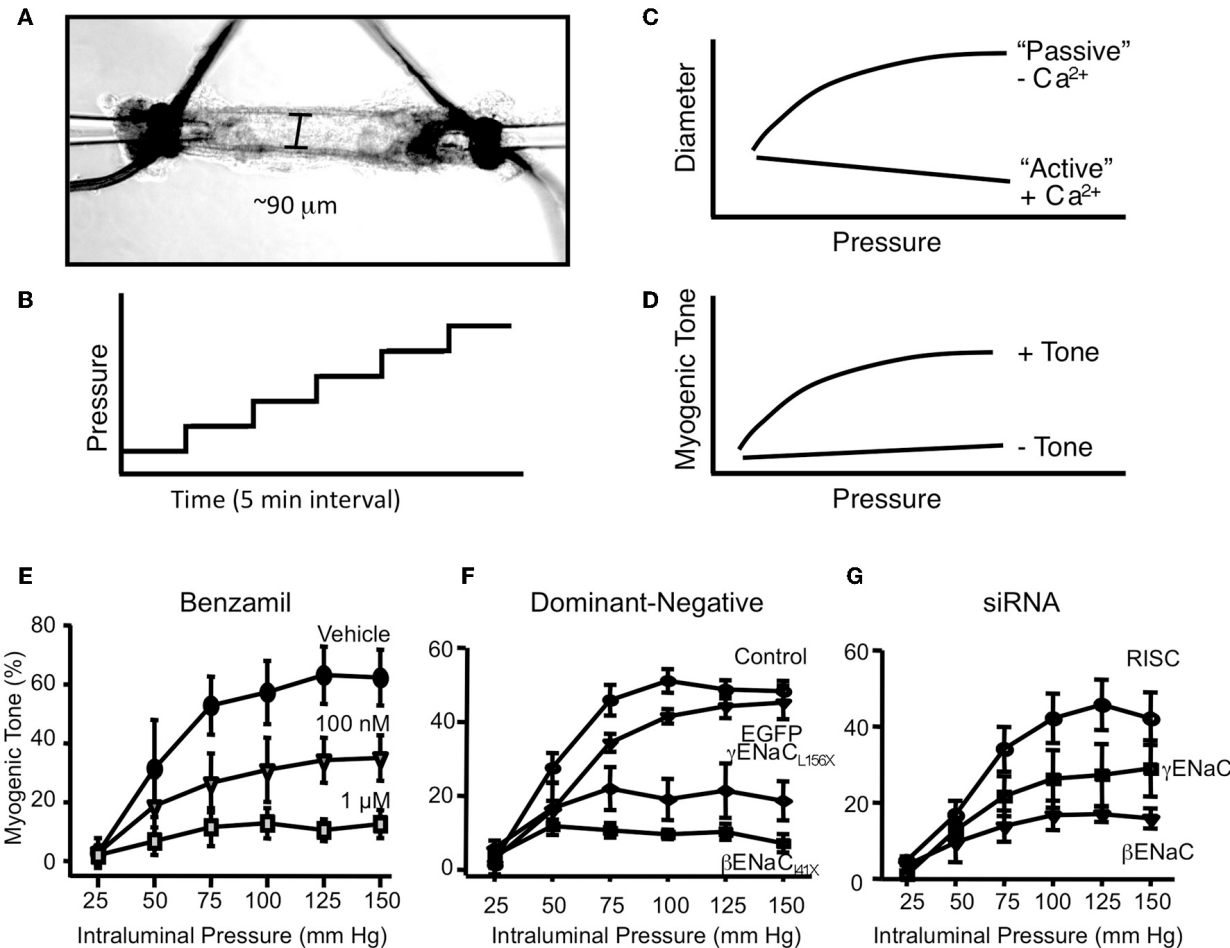
***In vitro* assessment of myogenic constriction. (A)** Image of an isolated renal interlobar artery segment tied to inflow and outflow perfusion pipettes. **(B)** To assess myogenic responsiveness vessels are exposed to a step-wise increase in perfusion pressure (25 mm Hg step, 5 min each) from 25 to 150 mm Hg under normal external Ca^2+^ and Ca^2+^ free solutions to determine active response and passive responses, respectively **(C)**. **(C)** The steady state diameter is plotted vs. pressure to obtain a pressure-diameter relationship under active vs. passive conditions. **(D)** Myogenic tone is calculated as [(passive diameter – active diameter)/passive diameter] at each pressure step. A vessel with active myogenic tone will have an increase in tone with an increase in pressure. A vessel with reduced or absent myogenic tone will have a flattened relationship between pressure and tone. **(E)** Effect of ENaC inhibition with benzamil on myogenic tone in renal interlobar arteries. **(F)** Transient gene-silencing of βENaC or γENaC using dominant-negative constructs of βENaC or γENaC inhibits myogenic tone in renal interlobar arteries. **(G)** Transient gene-silencing of βENaC or γENaC using siRNA inhibits myogenic tone in renal interlobar arteries. (Figure reproduced from American Journal of Physiology, Renal Physiology 289; F891-F901, 2005, Figure 4 and American Journal of Physiology, Renal Physiology 291; F1184–F1191, 2006, Figures 5, 6).

A second essential criterion to consider ENaC proteins as components of a mechanosensor in VSMCs is that physiological responses to mechanical activation of the myogenic response can be inhibited following ENaC blockade. Our laboratory has taken three approaches to determine the importance of ENaC proteins in renal myogenic constriction in renal interlobar arteries, which include (1) pharmacological inhibition, (2) transient gene silencing, and (3) genetically modified mice (Jernigan and Drummond, 2005, 2006; Ge et al., 2012). Our method for assessment of myogenic constriction is shown in Figures 3A–D. Renal interlobar artery segments are dissected from surrounding tissue and mounted on two pipettes (Figure 3A). Following an equilibration period to allow the vessel to develop spontaneous tone, we expose the vessel to a step-wise (25 mm Hg, 5 min) increase in perfusion pressure (Figure 3B), done during incubation with Ca^2+^ containing and then Ca^2+^ free extracellular solution. Under Ca^2+^ containing conditions, vessels will constrict in response to the increase in pressure. However, under Ca^2+^ free conditions, vessels will passively dilate in response to the increase in pressure (Figure 3C). Myogenic tone is calculated as the difference in diameter between Ca^2+^ containing and Ca^2+^ free conditions divided by Ca^2+^ free diameter. A vessel segment with a myogenic response will exhibit an increase in tone with an increase in pressure. The pressure-myogenic tone relationship will be flat in a vessel without a myogenic response (Figure 3D). If degenerin proteins are important in the transduction of myogenic constriction, then we expect the relationship between pressure and myogenic tone to be altered following degenerin inhibition.

To determine if degenerin proteins in general contributed to the transduction of the myogenic response, we first used broad-spectrum degenerin inhibitors amiloride and its analog benzamil (Jernigan and Drummond, 2005). As shown in Figure 3E, benzamil abolishes myogenic constriction in renal interlobar arteries in a concentration-dependent manner. Amiloride, data not shown, elicited a similar inhibition of myogenic constriction. An important factor in the interpretation of these experiments is the selectivity of the ENaC inhibitors. At submicromolar and low micromolar doses, benzamil is a fairly selective inhibitor of ENaC. Furthermore, recent studies by Guan et al. suggest myogenic constriction in rat afferent arterioles is also sensitive to ENaC inhibition (Guan et al., 2009). However, another study by Wang et al., benzamil/amiloride did not inhibit myogenic constriction in renal arterioles (Wang et al., 2008). While pharmacological inhibition is a great tool for screening for ENaC/ASIC involvement, specific subunit involvement cannot be determined. To determine the importance of the two ENaC proteins detected in renal VSMCs (βENaC and γENaC), we used siRNA and dominant-negative constructs to specifically silence β or γENaC expression in isolated mouse renal interlobar artery segments (Figures 3F,G) (Jernigan and Drummond, 2006). With these approaches, specific ENaC subunit expression was inhibited 50–70% in renal VSMCs. Following ENaC silencing, pressure-induced constrictor responses were inhibited by 40–80%, without altering the ability of the vessel to constrict to phenylephrine, suggesting the loss of vasoconstriction is specific to pressure rather than a generalized loss in the ability of the vessel to constrict.

## The βENaC m/m mouse: a model to determine the physiological importance of degenerin mediated myogenic constriction

Our early studies suggested an important role for certain degenerin proteins in myogenic constriction, however, understanding the consequences of long term loss of degenerin mediated myogenic constriction on cardiovascular health required a genetic model. For these studies, we focused on βENaC because our previous findings suggested βENaC may play a more important role.

### The βENaC m/m model

**Figure 4:**
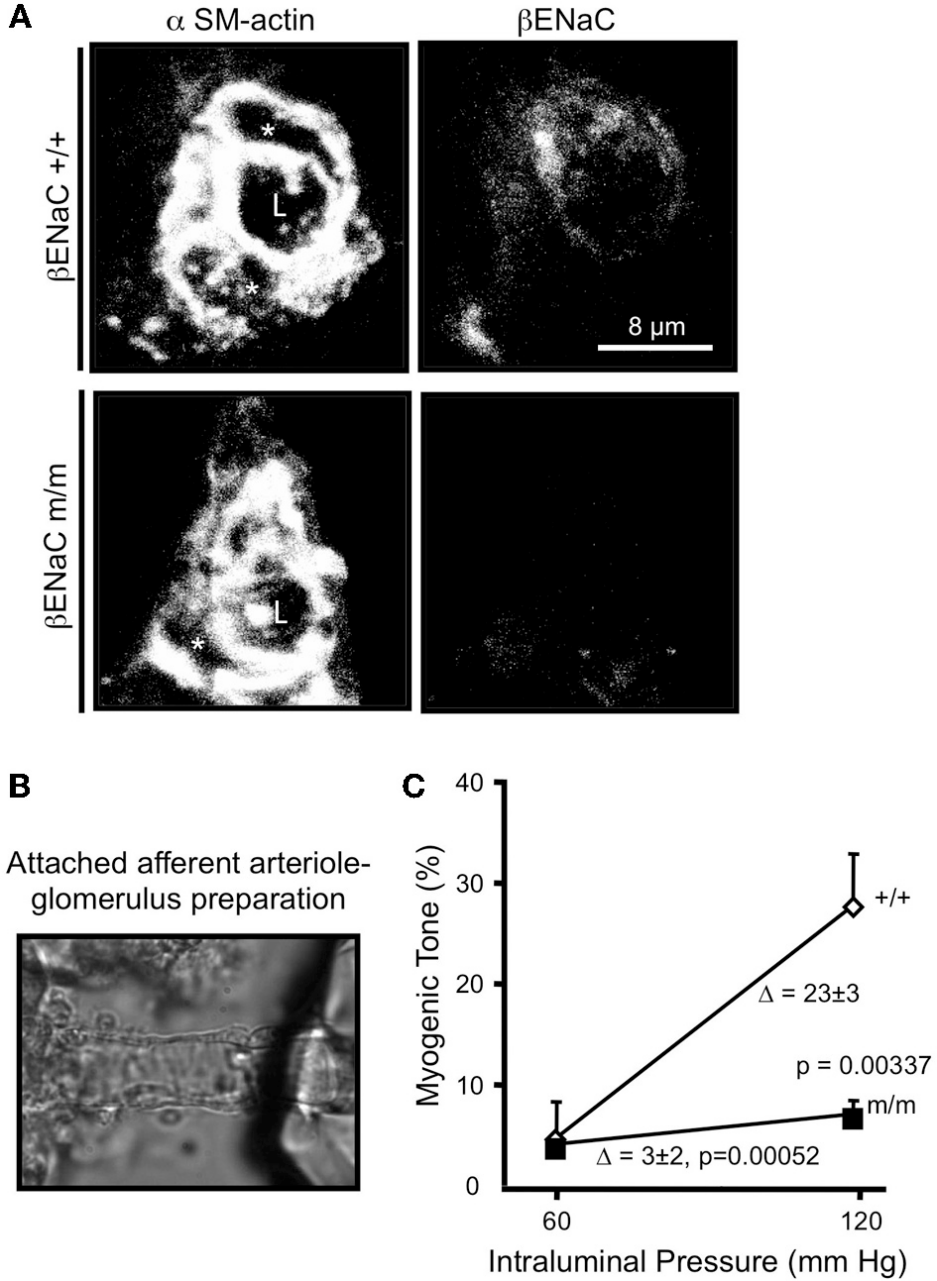
**Localization of βENaC and contribution to myogenic constriction in renal afferent arteriole. (A)** Localization of SM α-actin (left) and βENaC (right) in a cross-sectional image of a renal arteriole from a βENaC +/+ (top) and m/m (bottom) mouse. In the merged image, an “L” identifies the arteriolar lumen and an asterisk (^*^) identifies the VSMC cell bodies. **(B)** Image of an isolated afferent arteriole with attached glomerulus preparation. **(C)** Steady state vasoconstrictor responses to an increase in pressure in afferent arterioles in βENaC +/+ and m/m animals (*n* = 6). Vasoconstrictor responses to adrenergic agonist norepinephrine (NE) were similar (not shown). (Figure reproduced from American Journal of Physiology, Renal Physiology 302; F1486–F1493, 2012, Figures 1, 2).

Since βENaC null mice die shortly after birth (McDonald et al., 1999), we selected an alternative model with reduced levels of βENaC, βENaC m/m model developed by Bernard Rossier and Edith Hummler at the University of Lausanne, Switzerland (Pradervand et al., 1999). The βENaC m/m model was generated using standard gene targeting approaches in the course of generating a model of Liddle's syndrome (*increased* βENaC) by inserting a premature stop codon in the C-terminus coding region. However, the presence of the neomycin selection marker disrupts the βENaC gene locus resulting in *reduced* βENaC expression. Thus, a mouse model that under-, rather than over-expresses, βENaC was generated. Mice homozygous for the mutation (m/m) express very low levels of βENaC transcripts and/or protein in the lung, kidney, and VSMCs, including small cerebral arteries and renal afferent arterioles (Pradervand et al., 1999; VanLandingham et al., 2009; Grifoni et al., 2010; Drummond et al., 2011; Ge et al., 2012). Co-localization of βENaC with smooth muscle α-actin in afferent arteriolar VSMCs is shown in Figure 4A. Note the significantly reduced labeling for βENaC in renal afferent arteriolar VSMCs from βENaC m/m mice (Ge et al., 2012).

### Myogenic constriction in afferent arterioles is attenuated in βENaC m/m mice

To determine the importance of βENaC to afferent arteriolar myogenic constriction, we examined pressure-induced constriction using the attached afferent arteriole-glomerulus preparation (Figure 4B) (Ge et al., 2012). In this preparation, a single afferent arteriole attached to a glomerulus is dissected from the kidney and perfused at 60, then 120 mm Hg. The afferent arterioles from the βENaC +/+ mice develop significant myogenic tone in response to the step increase in pressure. However, afferent arterioles from βENaC m/m mice develop significantly less tone, suggesting βENaC mediates transduction of pressure-induced constriction in afferent arterioles (Figure 4C). This later finding is very significant because the afferent arteriole is the primary site of development of vascular resistance in the kidney, and thus likely contributes to control of whole kidney vascular resistance and blood flow regulation.

### Myogenic regulation of renal blood flow is attenuated in βENaC m/m mice

**Figure 5:**
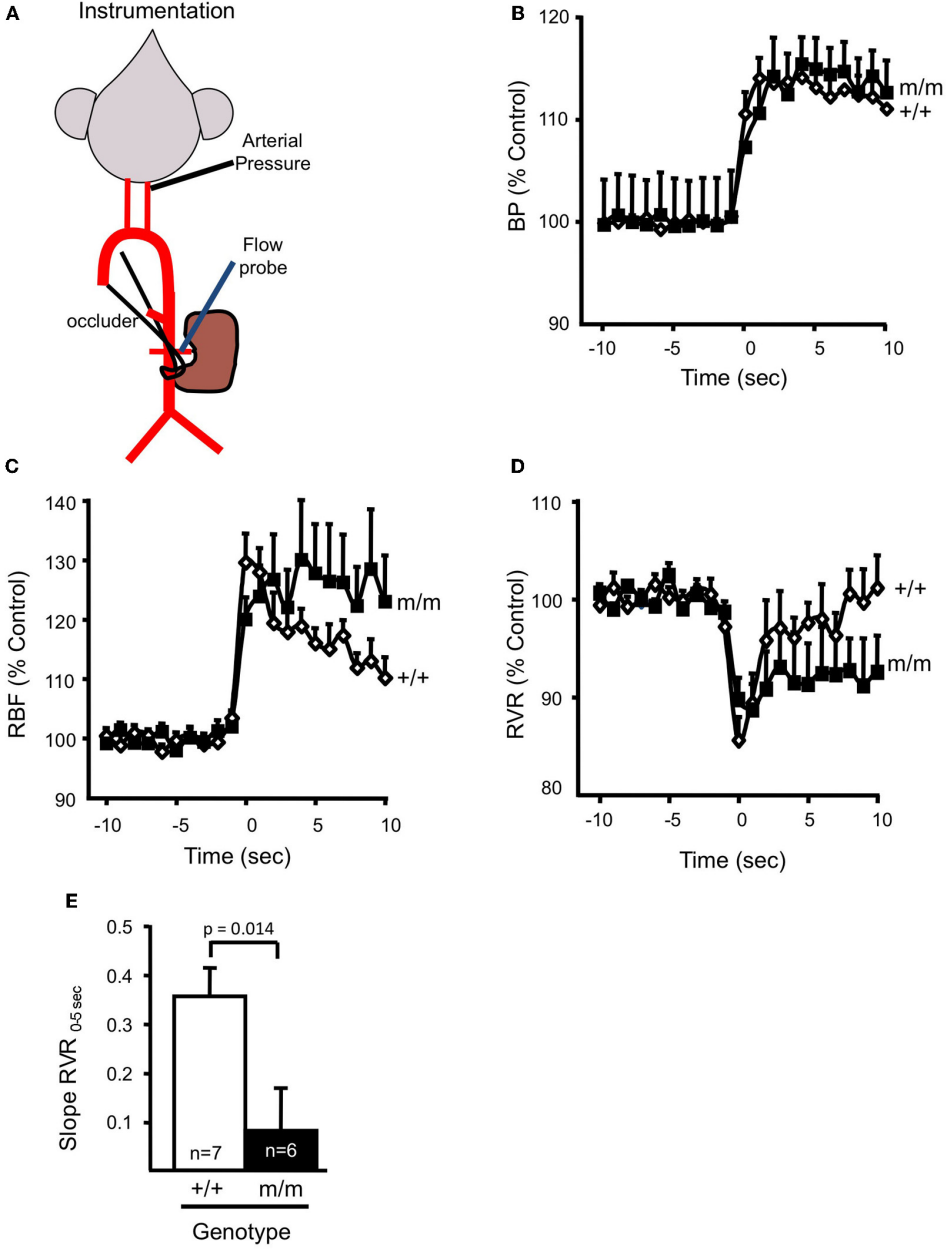
**βENaC mediates myogenic regulation of whole kidney blood flow and renal vascular resistance. (A)** Schematic of animal preparation. Mice have an arterial carotid catheter for blood pressure measurement and a renal flow probe for whole kidney blood flow measurement. An occluder tie placed around the lower abdominal aorta is used to generate the step increase in pressure. **(B)** Time course of the regulatory response of mean arterial pressure (MAP; **B**), renal blood flow (RBF; **C**), and renal vascular resistance (RVR; **D**), in wild type (+/+, filled symbols, *n* = 6) and mutant (m/m, open symbols, *n* = 7) 10 s before and 10 s after the step increase in pressure. Data are presented as normalized changes in **B**–**D** to minimize variance. Following a similar increase in MAP, the transient increase in RBF and decrease in RVR are similar between +/+ and m/m mice. Immediately following the transient drop, RVR begins to increase in the +/+, but remains low in the m/m. **(E)** The rate of increase in RVR during the first 5 s following the drop in RVR (Slope RVR_0–5 S_) is significantly greater in +/+ vs. m/m mice (*p* = 0.014). By 20–30 s following the step increase in MAP, RBF in the +/+ is corrected while RBF remains elevated in m/m animals. By 20–30 s following the step increase in MAP, the change in RVR from baseline is greater in the +/+ vs. m/m. Data are mean ± SEM. ^*^Significantly different from βENaC +/+ group at the *p*-value indicated. (Figure reproduced from American Journal of Physiology, Renal Physiology 302; F1486–F1493, 2012, Figure 3).

To begin to address the potential physiologic and pathophysiologic importance of βENaC mediated renal myogenic constriction in the regulation of whole kidney *renal* blood flow (RBF), we again turned to the βENaC m/m mouse model. In two recent studies, we utilized the temporal separation between the onset of the myogenic mechanism (0–5 s) and the TGF mechanism (6–25 s) to determine the contribution of βENaC to myogenic regulation of RBF (Grifoni et al., 2010; Ge et al., 2012) (Figure 5). In these studies, mice were instrumented with a carotid arterial catheter for blood pressure measurement and a renal flow probe for measurement of whole kidney blood flow (Figure 5A). A step increase in blood pressure was achieved with an occlusion of the lower abdominal aorta, just below the renal artery (Figures 5A,B). As shown in Figures 5C,D, RBF increases and RVR decreases immediately following the step increase in pressure. Within 5–10 s, RBF begins to return to control levels in +/+ animals due to myogenically mediated vasoconstriction (increase in RVR). In contrast, RBF remains elevated and RVR remains reduced in the m/m mice, suggesting a loss of myogenic regulation. To assess the integrity of renal myogenic regulation, we quantified the speed of the myogenic mechanism by the determining the rate of change in whole kidney vascular resistance during the first 5 s following a step increase in renal perfusion pressure [Slope RVR_0–5 S_, Figure 5E (Ge et al., 2012)]. We found myogenic speed was suppressed nearly 80%, findings that parallel our *in vitro* findings (loss of myogenic constriction in afferent arterioles) in βENaC m/m mice. Thus, the loss of βENaC mediated myogenic constriction prevents the rapid correction of RBF that normally occurs following a fluctuation in systemic blood pressure, thus leading to inappropriate regulation of RBF.

### Signs of renal inflammation and mild injury in the βENaC m/m mouse

**Figure 6:**
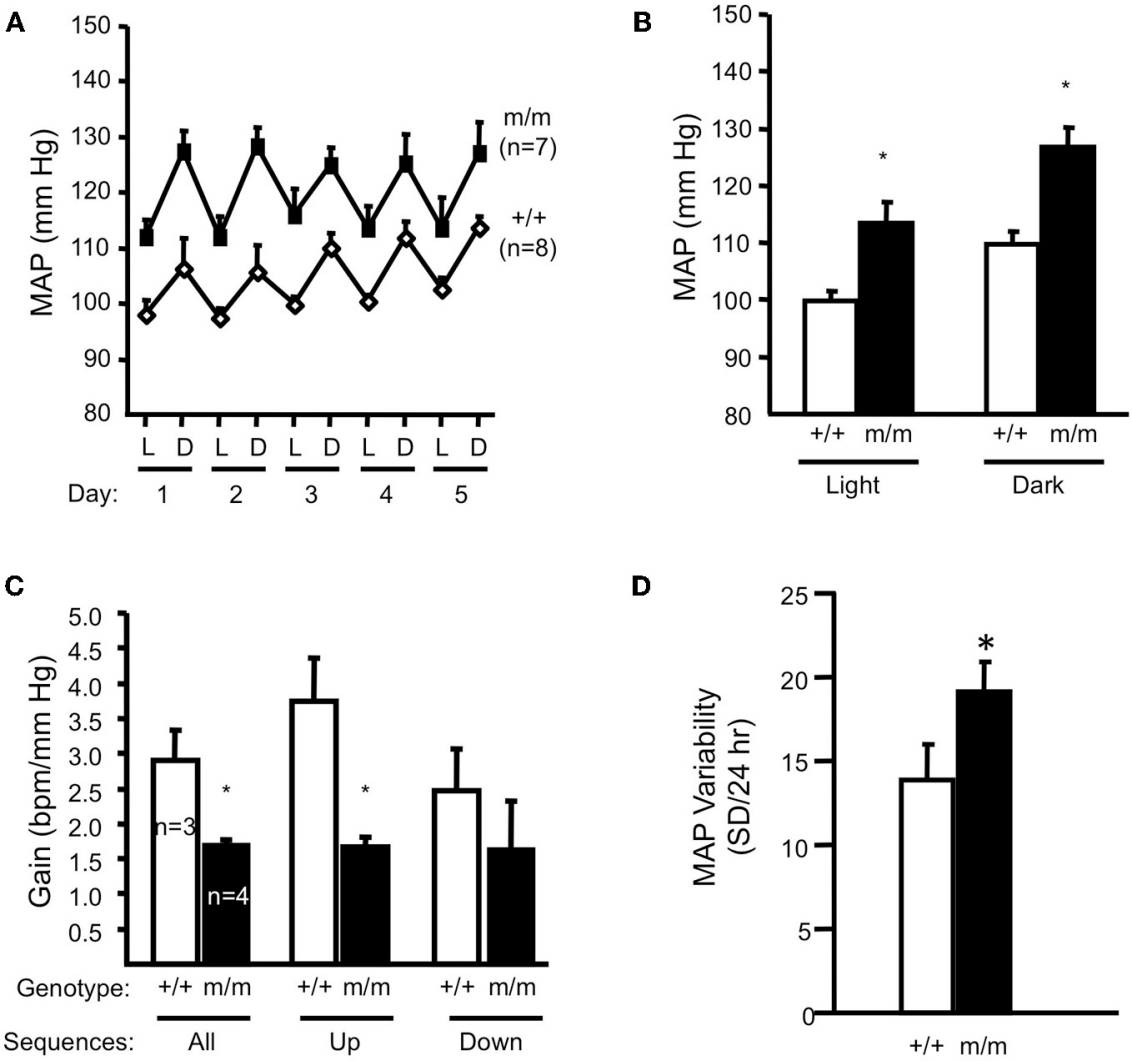
**Blood pressure and arterial baroreflex gain in normal salt (0.4% Na^+^) fed animals.** Mean arterial pressure (MAP) for 12-h light (L)–dark (D) cycles for each of 5 days and the average of 5 days are shown in panels **A** and **B**, respectively in wildtype (+/+, *n* = 8) and homozygous βENaC mutant mice (m/m, *n* = 7). **(C)** Arterial baroreflex gain is significantly lower in βENaC m/m mice (*n* = 4) vs. +/+ controls. **(D)** Mean arterial blood pressure (MAP) variability during the last 24 h of blood pressure recording was significantly elevated in βENaC m/m mice (same animals as shown in **A** and **B**). (Figure reproduced from American Journal of Physiology, Renal Physiology 301; F443–F449, 2011, Figures 2, 3). Data are mean ± SEM. BPM is heart rate in beats per minute. ^*^Significantly different from wildtype (+/+) control animals, *p* < 0.05.

Inappropriate regulation of RBF is linked to renal injury in hypertension and diabetes (Bidani et al., 2003, 2007). Normally, swings in systemic pressure are prevented from reaching the tiny, delicate renal microvasculature because of autoregulatory mechanisms; when systemic pressure rises, autoregulatory mechanisms are activated leading to vasoconstriction, which prevents transmission of higher systemic pressures to delicate microvessels (Bidani et al., 2003, 2007; Griffin et al., 2004; Loutzenhiser et al., 2006). The myogenic response responds rapidly to changes in perfusion pressure and is thus considered the principal mechanism to prevent transmission of pressure swings to the microvasculature. Since the βENaC m/m mice have reduced myogenic capacity, we considered the possibility that βENaC m/m mice might have signs of renal injury and possibly, *elevated* blood pressure. To address these issues, we examined kidneys for indicators of renal injury (Drummond et al., 2011). We found that compared to +/+ littermates, βENaC m/m mice have signs of inflammation and mild renal injury characterized by increased levels of renal inflammatory cytokines (TNFα, IL1β, IL6), inflammatory cells (macrophages, lymphocytes), growth factors linked to pressure-dependent injury (TGFβ), and mild expansion of extracellular matrix (Drummond et al., 2011). We also found that βENaC m/m mice have a mean blood pressure that is ~15 mm Hg higher than wildtype littermates (Figures 6A,B), as determined by telemetry (Drummond et al., 2011). These findings demonstrate a link between altered βENaC mediated myogenic function, renal injury, and hypertension.

### Isn't the βENaC m/m mouse supposed to be hypotensive?

Because the βENaC m/m mouse was generated using homologous recombination, reduced levels of βENaC would be expected in all tissues, including renal tubular cells. Loss of tubular ENaC related salt and water transport would be expected to be associated with reduced or normal blood pressure (with compensatory up-regulation of sodium retaining hormones). Thus, at first consideration, our finding that blood pressure is elevated in the βENaC m/m may seem counter-intuitive. However, when the elevated blood pressure data is taken in context with loss of myogenic autoregulation and presence of renal inflammation in the βENaC m/m, renal injury related increases in blood pressure becomes a plausible explanation.

### Contributing factor to renal injury: arterial baroreceptor dysfunction in βENaC m/m mice?

**Figure 7:**
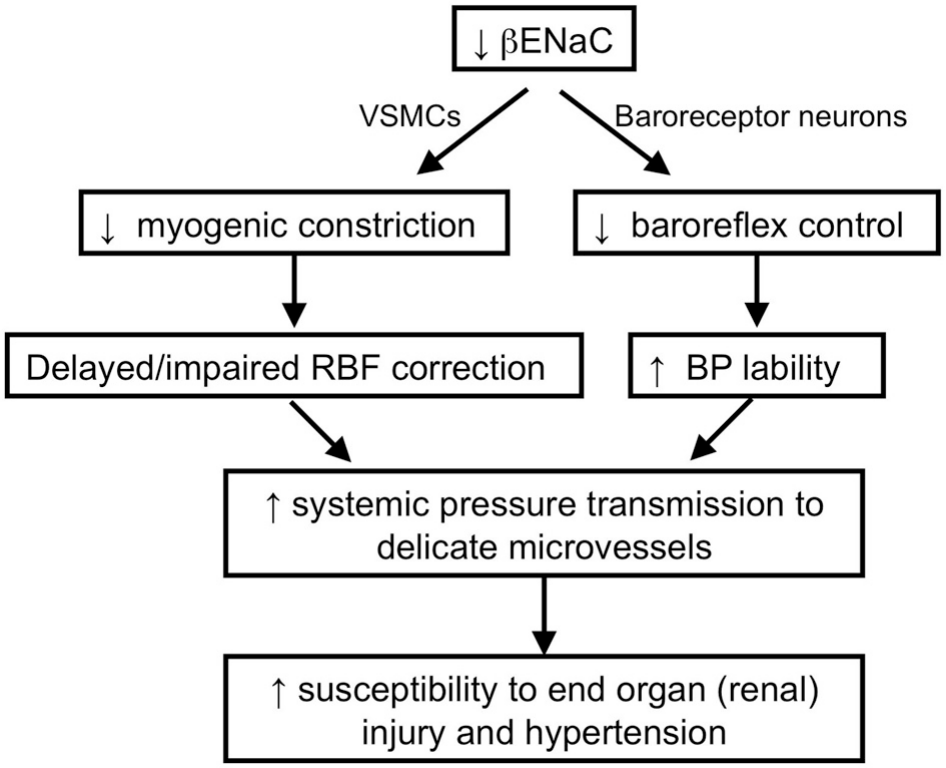
**Schematic of working hypothesis: the role of βENaC as a mechanosensor and its role in cardiovascular pathophysiology.** We hypothesize that βENaC is a critical component of mechanosensors in renal VSMCs and arterial baroreceptor neurons. Loss in βENaC function in renal VSMCs leads to a loss in renal myogenic constriction and a delayed or impaired correction of renal blood flow following upward swings in blood pressure. Loss in βENaC function in arterial baroreceptor neurons leads to a loss in baroreflex control of blood pressure and increased BP lability. Increased BP lability and decreased myogenic constriction increase the opportunities and magnitude of systemic pressure transmission to the renal microvessels, which in turn, increases susceptibility to end-organ injury and hypertension.

ENaC proteins have also been considered potential mechanotransducers in arterial baroreceptors, which are the sensory portion of a neural reflex that helps to buffer fluctuations in arterial blood pressure that occur with normal activity (Cowley et al., 1973; Drummond et al., 2001). When blood pressure suddenly changes, specialized nerve endings located in the aortic arch and carotid sinuses senses changes in vascular stretch and relay signals to the brain stem to inversely control sympathetic and parasympathetic nerve activity to periphery to adjust vascular resistance, heart rate and cardiac contractility to return blood pressure to control. For example, a sudden increase in blood pressure will activate peripheral arterial baroreceptors, which will send signals to the brain stem. In response, sympathetic outflow will be suppressed to decrease cardiac contractility, heart rate, and vascular resistance and return blood pressure to control. If βENaC were a component of an arterial baroreceptor mechanotransducer, then we would expect that βENaC m/m mice might have altered baroreflex sensitivity. During collection of blood pressure data in the mice, we noticed an increased variability in blood pressure values among measurement intervals in βENaC m/m mice (Drummond et al., 2011), suggesting a labile blood pressure consistent with a loss of baroreceptor function. To further address the possibility that arterial baroreceptor function may be altered in βENaC m/m mice, we used “sequence analysis” to determine if heart rate responses to spontaneous fluctuations in systemic blood pressure were altered in βENaC m/m mice (Figures 6A,B). For these studies, we used the approach and criteria used by others (Braga et al., 2008). We found that gain of the baroreceptor sequences (up and down) were significantly lower in βENaC m/m mice (Figure 6C), consistent with reduced baroreflex sensitivity. This is an important finding because altered baroreflex sensitivity would be expected to increase blood pressure variability, which would increase the magnitude and/or frequency of blood pressure swings. To determine if the altered baroreflex sensitivity translated into increased variability in blood pressure, we quantified the variability in mean arterial pressure (MAP) measurements during the last 24 h of blood pressure recording. As shown in Figure 6D, MAP was more variable in βENaC m/m mice. Normally, these blood pressure swings should not pose a problem, however, if these swings are superimposed on an background of weakened myogenic constriction, the increased frequency and/or magnitude of upward swings in blood pressure translate may into more opportunities for transmission of damaging systemic pressure to the delicate renal microvasculature, and consequently, increased likelihood for renal injury (Figure 7). Over the course of a lifetime, those repeated pressure insults to the microvessels are likely to promote injury. Thus, reduced baroreflex sensitivity, coupled with reduced myogenic capacity, likely contributes to the renal injury and increased blood pressure observed in the βENaC m/m model. This hypothesis has been proposed previously for another model of hypertensive renal injury (Iliescu, 2007).

## Future directions

Degenerin mediated vascular function is a new field, and as such, there is much that needs to be done to elucidate the importance of degenerins in vascular function. Pressure-dependent renal injury is a leading cause of end-stage renal disease with substantial financial costs (Medicare Care costs exceeded $26 billion in 2010) (USRDS, 2008, 2010). Understanding the degenerin mediated protection from injury will lead to development of approaches to prevent renal injury. Although the loss of renal pressure-dependent vascular function in βENaC m/m mice does not lead to *severe* renal injury and hypertension, it is not clear if a “second hit,” such as high Na^+^ diet, elevated angiotensin II, or added psychological stress would increase the severity of injury. Furthermore, information learned from the renal circulation may also apply to other myogenically active circulations, such as the cardiac and cerebral beds.

Determining the identity of other proteins that form the heteromultimeric mechanosensor in VSMCs is also an important future direction. Based on the *C. elegans* model, the mechanosensor is a large heteromeric complex. The pore, which may be formed one or more degenerin proteins, is tethered to the cytoskeleton and extracellular matrix. The identity of the other pore forming subunit(s) and the cytoskeleton and extracellular matrix proteins responsible for tethering the pore in VSMCs have not been identified. Understanding their identity and regulation by hormonal, inflammatory, and autocrine factors may provide additional insight into the prevention and treatment of renal injury. It is very likely that VSMC ENaC protein expression and/or function may be altered in hypertension because many of the “usual suspects” implicated in hypertension (i.e., endothelin, aldosterone, angiotensin II, inflammatory cytokines, reactive oxygen species, and nitric oxide, dietary salt) regulate tubular ENaC and neuronal ASIC channel expression (Gilmore et al., 2001; Mamet et al., 2002, 2003; Beutler et al., 2003; Amasheh et al., 2004; Barmeyer et al., 2004; Drummond et al., 2006; Helms et al., 2008; Jernigan et al., 2008, 2009; Pavlov et al., 2010; Chung et al., 2011).

## Summary

Our laboratory has considered degenerin proteins, specifically βENaC and γENaC, as components of a mechanosensor in VSMCs because of their strong evolutionary link to mechanotransduction in *C. elegans*. Multiple lines of *in vitro* and *in vivo* evidence support this hypothesis. The evidence includes (1) appropriate protein localization, (2) disruption of myogenic constriction in isolated vessels using pharmacological, transient gene silencing, and genetically modified animals, and (3) disruption of myogenically mediated whole organ blood flow *in vivo*. Furthermore, the potential importance of degenerin mediated vascular function on cardiovascular health is becoming clear; loss of vascular degenerin function may inhibit the protective renal myogenic mechanism and increase blood pressure variability, thereby increasing susceptibility to pressure related renal injury and hypertension.

### Conflict of interest statement

The author declares that the research was conducted in the absence of any commercial or financial relationships that could be construed as a potential conflict of interest.
